# Eye Drops of Metformin Prevents Fibrosis After Glaucoma Filtration Surgery in Rats *via* Activating AMPK/Nrf2 Signaling Pathway

**DOI:** 10.3389/fphar.2020.01038

**Published:** 2020-07-31

**Authors:** Xueru Li, Yu Leng, Qingzhi Jiang, Ziwen Wang, Peng Luo, Chi Zhang, Long Chen, Yawei Wang, Huilan Wang, Xiaofeng Yue, Chongxing Shen, Yuanlinhan Zhou, Chunmeng Shi, Lin Xie

**Affiliations:** ^1^ Department of Ophthalmology, The Third Affiliated Hospital of Chongqing Medical University (Gener Hospital), Chongqing, China; ^2^ Department of Oncology, The Affiliated Hospital of Southwest Medical University, Luzhou, China; ^3^ Institute of Rocket Force Medicine, State Key Laboratory of Trauma, Burns and Combined Injury, Third Military Medical University, Chongqing, China; ^4^ Department of Urology, The Third Affiliated Hospital of Chongqing Medical University (Gener Hospital), Chongqing, China; ^5^ Chongqing Industry Polytechnic College, Chongqing, China

**Keywords:** glaucoma filtration surgery (GFS), metformin, fibrosis, AMPK/Nrf2, oxidative stress, macrophages, organic cation transporters (OCTs)

## Abstract

Metformin has effective therapeutic effects in anti-tumor and anti-fibrotic diseases. However, how the antifibrotic effect of metformin in the eye and how it is transferred are still unclear. Here, the eye drop of metformin treatment was studied in Sprague–Dawley (SD) rats of glaucoma filtrating surgery (GFS). Rats were administered randomly bilateral drops: control group (without surgery), GFS group, metformin group or mitomycin C (MMC) group (sponge application intraoperatively, 0.02%). Bleb features and intraocular pressure (IOP) were assessed for postoperative week 4. Metformin effectively inhibited fibrosis and improved the surgical outcomes of GFS. *In vitro*, we found that the degree of oxidative stress and fibrosis in metformin pretreated-Human Conjunctival Fibroblasts (HConFs) were reduced; the pro-fibrotic response of HConFs were decreased by inducing macrophagic polarity changes. Besides, the inhibition of nuclear factor erythroid 2-related factor 2 (Nrf2)/AMP-activated protein kinase (AMPK) and the competition of organic cation transporters (OCTs) effectively reduced the anti-fibrotic capability of metformin. Together, this experiment indicates that metformin enters into HConFs cell with OCTs, which can protect against filtrating blebs scar formation in SD rats of GFS *via* activating AMPK/Nrf2 axis and the downregulation of profibrogenic and inflammatory biomarkers.

## Introduction

Glaucoma filtration surgery (GFS) is the most efficacious option for uncontrolled-IOP glaucoma besides medicine and laser treatment (Lanzl et al., 2016). However, with the destruction of the tissue vasculature, the stimulation of exudate and hormones on conjunctival fibroblasts, these are risk factors for pathological filtration of fibrotic fibrosis. Postoperative scar formation is the major obstacle to increased success (Zada et al., 2018; Addicks et al., 1983; Schlunck et al., 2016). Currently, anti-inflammatory medication and antimetabolites such as Mitomycin C (MMC), 5-fluorouracil (5-FU) and steroids are widely applied in clinical, but complications (corneal toxicity, hypotony, wound leak and even infectious endophthalmitis) limit their applications (Cabourne et al., 2015). Therefore, further improvements and strategies are urgently needed to better control filtrating blebs fibrosis.

Recently, accumulated literatures reported that the therapeutic effects of metformin in anti-tumor and anti-fibrotic diseases such as breast cancer (Patterson et al., 2018; Sharma and Kumar, 2018), infertility (Sivalingam et al., 2014) and KRAS/LKB1 co-mutated NSCLC (Moro et al., 2018) and fibrosis (Biondo et al., 2018; Juban et al., 2018). Metformin exhibited pleiotropic mechanisms for cell protection including mitochondrial integrity, proliferation, inflammatory response, oxidative stress and ER stress (Sato et al., 2016; Sharma and Kumar, 2017). Moreover, metformin is an AMPK activator as a crucial sensor of cellular bioenergy, which can control the switch from anabolic to catabolic. It was resistant to stress and maintained the balance of cellular metabolism when sensing and decomposing mitochondrial ROS. (Hardie et al., 2012; Quarta et al., 2019), which were risk factors for pathologic fibrotic processes (Salminen and Kaarniranta, 2012). Not only that effects of oral administration of metformin on ocular disease have also attracted widespread attention, the preclinical studies showed that metformin exerted protective effects on reducing risks of developing open angle glaucoma (OAG) (Lin et al., 2015) and had obvious anti-inflammatory and anti-angiogenic effects on retinal vascular hyperplasia (Han et al., 2018b). However, it is not clear how the antifibrotic effect of metformin in the eye and how it is transferred.

In this study, the experimental results showed that eye drops of metformin could effectively reduce fibrosis and prolonged bleb survival in SD rats GFS models postoperative 4 weeks. Mechanistically, metformin entered into Human Conjunctival Fibroblasts (HConFs) by organic cation transporters (OCTs), which reduced HConFs fibrosis *via* a mechanism involving oxidative stress promoting and anti-fibrosis in TGF-β2-induced collagen production. Our results highlight the potential of using metformin eye drops to treat patients after GFS.

## Methods and Materials

### Reagents and Antibodies

The TGF-β2 cytokine was purchased from Pepro Tech. Glyceraldehyde-3-phosphatedehydrogenase (GAPDH), horseradish peroxidase (HRP)-conjugated secondary antibodies, RIPA buffer, H2DCF-DA, BCA kit, western blocking buffer, H_2_DCF-DA were the products of Beyotime (China). The nucleoside 5-aminoimidazole-4-carboxamide riboside (AICAR), metformin hydrochloride, Phorbol 12-myristate 13-acetate (PMA), ML385 (an Nrf2 inhibitor) (CAS No.: 846557-71-9), Atropine (CAS No.: 51-55-8) and Quinidine (CAS No.: 56-54-2) were acquired from MedChemExpress (MCE) (China). Antibodies against AKT, p-AKT, AMPK and p-AMPK (172) were purchased from CST. Antibodies against superoxide dismutase 1 (SOD1), SOD2, γ-glutamyl cysteine synthetase (γ-GCS), Catalase (CAT), smad2/3 and β-actin were purchased from Proteintech. Antibodies against Nrf2, α-smooth muscle actin (α-SMA) (ab5694), Osteopontin (OPN) (ab8448), collagen-I, p-smad2/3 and fibronectin (ab34710) were purchased from Abcam. Antibody against p-Nrf2(S40) was provided by Santa Cruz Biotechnology. The cell culture reagents were the products of Gibco Laboratories (CAS No.:16000-044).

### Cell Culture

Primary HConFs (or THP1) cell lines were acquired form ATCC (USA) for this study. The HConFs (or THP1) was cultured in DMEM (or RIPM 1640) containing 10% FBS (Gibco, USA), 1% cyan-streptomycin at 37°C with 5% CO_2_. The serum-starved (12 h) HConFs was used for cell experiments.

### Rat Glaucoma Filtration Surgery Models and Treatments

Thirty-two female SD rats (6–8 weeks, 200–250 g) were used. The animals were anaesthetized by intraperitoneal injection of overdose 10% Chloral Hydrate (1 ml/100 g). Topical anesthesia (0.5% oxybutyn hydrochloride) was used before the operation. GFS was performed as previously described (Sherwood et al., 2004). A conjunctival flap based on the limbus was formed 3–5 mm behind the limbus. Then, inserted a 25G needle into anterior chamber (AC) to create a full-thickness scleral tunnel. Afterwards, a beveled 30-G microcannula was inserted into the scleral tunnel. The microcannula was fixed to the limbus. Finally, 10-0 (0.1 metric) Ethicon monofilament nylon sutures were used to close the conjunctiva. Random bilateral drug administration to rats: control group (without surgery), GFS group (saline, with surgery), metformin group (7.85 mM/drop, four times/day and postoperative 2 weeks) (N = 5) and MMC group (sponge application, 0.02%), which were evaluated at a pre-set endpoints.

### Histological Examinations

On postoperative 14 days, twenty female SD rats were euthanized. Removing their surgical eyeball, A small opening was cut away from surgical wounds, then soaked the eyeball in 4% formaldehyde solution (48 h). The tissues were dehydrated, encapsulated, sliced, and then used for H&E and Masson trichrome staining. Tissue sections were also used for immunofluorescence staining. The sections were dewaxed, rehydrated, and treated with 3% hydrogen peroxide at room temperature for 10 min. Then, the sections were treated with 0.4% pepsin at 37°C for 30 min to recover the antigen, followed by blocking with 1% goat serum albumin at room temperature for 40 min (Li et al., 2020). After incubating with primary antibodies(α-SMA, vimentin and type 1 collagen, 1:200) at 4°C overnight, the sections were incubated with an appropriate biotinylated secondary antibody at 37°C for 1 h, and the image was captured under an optical microscope (Olympus Inc., Tokyo, Japan).

### Transwell Co-Culture Assay

The bottom membrane of the Trans-well chamber was coated with serum (37°C, 1 h), followed by washing with PBS. Collected THP1 cells in logarithmic phase, adjusted the cell density to 1 × 10^5^ cells/ml with 10% FBS medium, added 500 μl of cell suspension to the lower chamber of the 24-well plate, and induced with PMA (100 nM) for two days. On third day, macrophages M1, M2 different inducers LPS, IL-4 + IL-13 were added and cultured for 24 h under the intervention of metformin (2 mM). The HConFs (1 × 10^5^ cells) was cultured (serum-free medium) in a 24-well plate compartment and co-cultured for different time periods. Removed the trans-well chamber, discard the culture medium in the wells, gently wiped the upper non-migrating cells with a cotton swab, fixed with paraformaldehyde fixative (20 min) followed by washing with water carefully after staining with crystal violet for 20 min. Cells were randomly observed in five fields under a 400× microscope and counted.

### Western Blotting Analysis

Total protein was extracted and quantified by BCA kit. Transferred protein of different molecular weight to polyvinylidene fluoride membrane by SDS-PAGE (Millipore) followed by blocking with blocking buffer (37°C, 1 h). The membranes were incubated with a primary antibody (γ-GCS, SOD1/2, CAT, p-Nrf2 (S40), Nrf2, smad2/3, p-smad2/3, vimentin, α-SMA, fibronectin, Akt/p-Akt, AMPK/-AMPK, collagen 1 (ECM deposition level) and GAPDH, 1:1,000) overnight at 4 °C. At room temperature, the blots and HRP-conjugated secondary antibody (CST, 1:1,000) were washed three times (15 min/times) with TBST at the beginning and end of the 1-hour incubation on a shaker. An enhanced chemiluminescence detection system (Bio-Rad Laboratories) and Image J software (Bethesda, Maryland, USA) were used, the intensity of bands normalized to GADPH statistics was observed and analyzed (Li et al., 2020).

### Real-Time PCR

We isolated total mRNA from HConFs cell with TRIzol^®^ reagent. A Prime Script TMRT kit with cDNA Eraser (#K1622) was used for reverse transcription. qRT-PCR was performed by SYBR Green qPCR master mix (Takara, Japan). The total reaction volume was 20 μl = 10 μl (SYBR solution, 2.5× Real Master Mix) + 0.5 μl (Forward primer,10 μmol/L) + 0.5 μl (Reverse primer, 10 μmol/L) + 1 μl cDNA + 8 μl (ultra-pure water) (Li et al., 2020). Reaction conditions: Total mRNA was pre-denatured at 95 °C for 10 min, denatured at 95 °C for 15 s, annealed at 62 °C for 30 s, extended at 68 °C for 30 s, and 50 cycles. The 2^−ΔΔCt^ method was used to calculating gene levels and normalize relative to GAPDH (Li et al., 2020).

### Cell Viability Assay

The cells suspension was inoculated into 96-well plates (4 × 10^3^ cells/well) and preconditioned by TGF-β2 with appear or without appear metformin (2 mM) treatment for 24, 48 and 72 h. Then, HConFs were cultured in 100 μl fresh complete medium (48 h) and then added 10 μl CCK-8 reagent/well. After incubating for 1 h at 37°C, the absorbance values were measured with a microplate reader (Multiskan Go Multimode Reader, Thermo Scientific).

### Measurement of Reactive Oxygen Species

According to the previous experimental method (Li et al., 2020), to assess the oxidative stress level of HConFs, cells were planted into 6-well plates (4 × 10^4^ cells^−1^). After different treatment, the wells were decanted and washed two times. A 500 μl reaction solution H2DCF-DA (5 × 10^−6^ m, 1:1,000)/MitoSOX Red (5 μM, CAS No.:M36008) for mitochondrial ROS evaluation was incubated at 37 °C for 20 min in the dark. Next, trypsin-digested-HConFs were resuspended in 150 μl with PBS. Then the signaling was tested by polychromatic flow cytometry and the data were analyzed (Flow-Jo_V10 software).

### Confocal Microscopy Imaging

HConFs cells were seeded on 14 mm glass coverslips of 24-well plates and incubated for 24 h after different treatments. Slides were washed (two times) and fixed in 4% paraformaldehyde solution at 4°C overnight. It was blocked with 1% goat serum (1 h, 37°C) after infiltration with 0.1% Triton-X100 (Li et al., 2020). Primary antibody fibronectin (1:200) was incubated at 4°C overnight. Next, the slides were washed with PBS and incubated with AlexaFluor^®^594/AlexaFluor^®^488-conjugated secondary antibody (1:500) for 40 min at 37°C in the dark followed by DAPI staining (2 min). A fluorescence microscope (laser scanning confocal microscope, Leica) was used to capture the images and analyze them.

### Co-Culture Analysis

To analyze the role of macrophages of different polarities on fibrosis of HConFs cells under the intervention of metformin (2 mM). Collected THP1 cells in logarithmic phase, adjust the cell density to 1 × 10^5^ cells/ml with 10% FBS medium, added 2 mL of cell suspension to the higher chamber of the 6-well plates, and induced with PMA (100 nM) for two days. On third day, M0 macrophage was induced by LPS or IL-4/IL-13 (100 ng/ml or 20 ng/ml) into macrophages M1, M2 and cultured for 24 h under the intervention of metformin (2 mM), and then the HConFs (1 × 10^5^ cells) were treated with serum-free medium in a 6-well plates compartment and were co-cultured after different time periods. Collected the HConFs in the lower chamber of the 6-well plates, and performed qRT-PCR/western blotting experiments to detect the fibrosis and proliferation levels of HConFs cells.

### Statistical Analysis

All results were presented as mean ± standard deviation. One-way analysis of variance was applied to determine signiﬁcance among groups. Statistical signiﬁcance was set at ^*/#^p <0.05, ^**/##^p <0.05 and ^**/###^p <0.01. All the statistical analyses were performed using SPSS13.0 software (SPSS, USA).

## Results

### Metformin Efficiently Prolongs Filtering Bleb Survival in Rats of GFS

**Figure 1:**
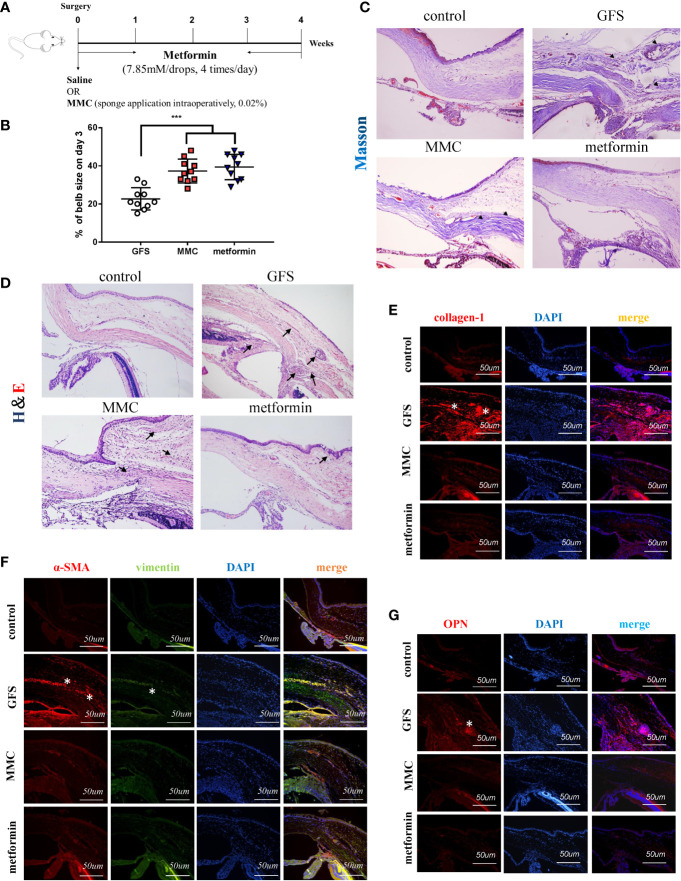
Metformin prolongs filtering blebs survival in SD rats of GFS. **(A)** Schematic illustration of the experimental animal timeline. **(B)** Quantization of bleb area was analyzed. **(C)** H&E and **(D)** Masson’s trichrome were assessed for the fibrotic levels of filtrating blebs. **(E**–**G)** Immunohistochemical staining images showing the expression of collagen-1, vimentin, α-SMA and OPN in filtrating bleb tissues at postoperative day 14. n = 10. Data shown as mean values ±  SD. *** denotes p < 0.001.

Accumulated studies reported that metformin has therapeutic effects on non-diabetic diseases. However, the antifibrotic role of metformin in ocular diseases is not clear. In this study, the role of topical metformin treatment was studied in SD rats of GFS (**Figure 1A**). The slit lamp inspection was to assess typical appearances of bubbles in SD rats’ model at the 3, 7, 14 and 28 days postoperative endpoints (**Supplemental Figure S1A**). Representative images showed that metformin group (7.85 mM/drops (an average of 30 ul/drop), four times/day) significantly prolonged bleb survival. But the bubbles were flat, small and vascularized in GFS group. There were lager areas of functional filtration blebs in metformin group (**Figure 1B**). There is a higher intraocular pressure (IOP) in metformin group than MMC group and GFS group on 28 day postoperatively (**Supplemental Figure S1B**). Moreover, H&E/Masson’s trichrome staining were used to assess tissue sections. Representative images revealed that the collagen deposition in metformin group was significantly decreased than GFS group (**Figures 1C, D** **)** (as black arrows showed). Furthermore, Masson’s trichrome staining of surgical eyes in GFS group almost showed server scars at the surgical site, including more evidences of collagen deposition in surgical sites than MMC/metformin treatment. α-SMA, vimentin, collagen-1, OPN, fibronectin and CD31 were expressed higher levels in GFS group than metformin group with immunofluorescence staining (**Figures 1E, F** and **Supplemental Figures S1D, E**) (as white stars showed). Metformin significantly reduced the protein expression of OPN compared to GFS group, suggesting that the existence of lighter inflammatory response at surgery sites (**Figure 1G**) (as white stars showed). Therefore, the above results indicate that metformin reduces fibrosis in rats of GFS.

### AMPK Is Critical for Fibrosis in TGF-β2-Induced HConFs

**Figure 2:**
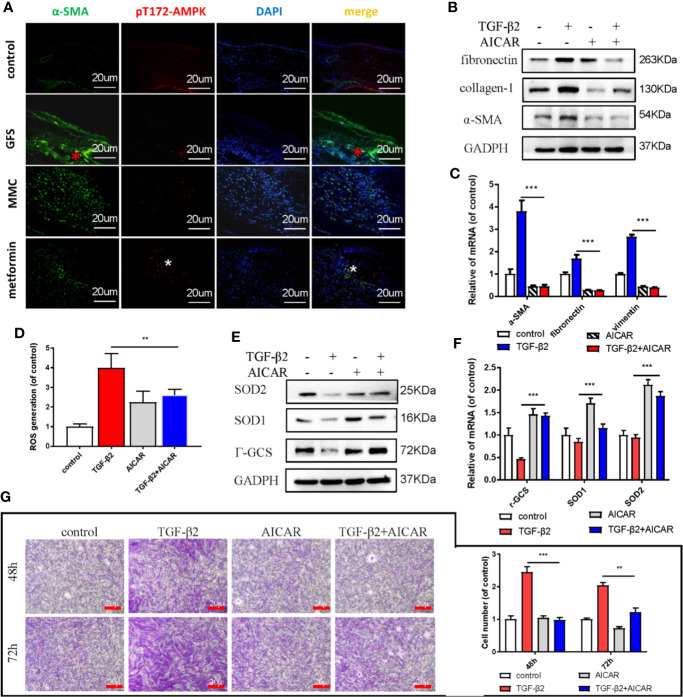
AICAR inhibits TGF-β2-induced HConFs fibrosis. **(A)** The expression of Thr172 AMPK phosphorylation in filtrating blebs tissues in rats were analyzed by historical images. **(B**, **C)** The fibrotic levels in TGF-β2 treated HConFs followed by AICAR treatment were determined by western blotting/qRT-PCR. **(D)** The ROS levels were analyzed by flow cytometry assay. **(E)** Relative antioxidants proteins and **(F)** genes were determined. **(G)** The migration capabilities of HConFs were assessed by transwells. n = 3. All data shown as mean values ± SD. ** and *** denote p < 0.01 and p < 0.001, respectively.

AMPK, a key sensor of cellular bioenergy, can control the conversion from anabolism to catabolism. Besides, AMPK is a known critical factor for modulating the pathological fibrosis process in terms of sensing and solving the resistance of mitochondrial ROS and maintaining cellular metabolic balance (Park et al., 2012; Rangarajan et al., 2018). Postoperative 2 weeks in SD rats GFS model, the immunohistochemical images showed that the level of α-SMA at the wound site (GFS group) was significantly increased, but with a lower activated level of AMPK, as evidenced by decreased the phosphorylated level of Thr172 AMPK (**Figure 2A**) (as red/white stars showed). AICAR, an AMPK activator, prevented collagen-I, fibronectin, and α-SMA expressions in TGF-β2 (4 ng/ml) treated HConFs cells (**Figures 2B, C** **)**. Besides, flow cytometry results showed that AICAR effectively reduced TGF-β2-induced the increase of ROS (**Figure 2D**). Meanwhile, AICAR increased the levels of antioxidants relative proteins/genes of γ-GCS, SOD1, SOD2 (**Figures 2E, F** **)** and significantly reduced the migration in HConFs after TGF-β2 pretreatment (**Figure 2G**). The above results indicate that AICAR effectively inhibit TGF-β2-induced cell proliferation and oxidative stress.

**Figure 3:**
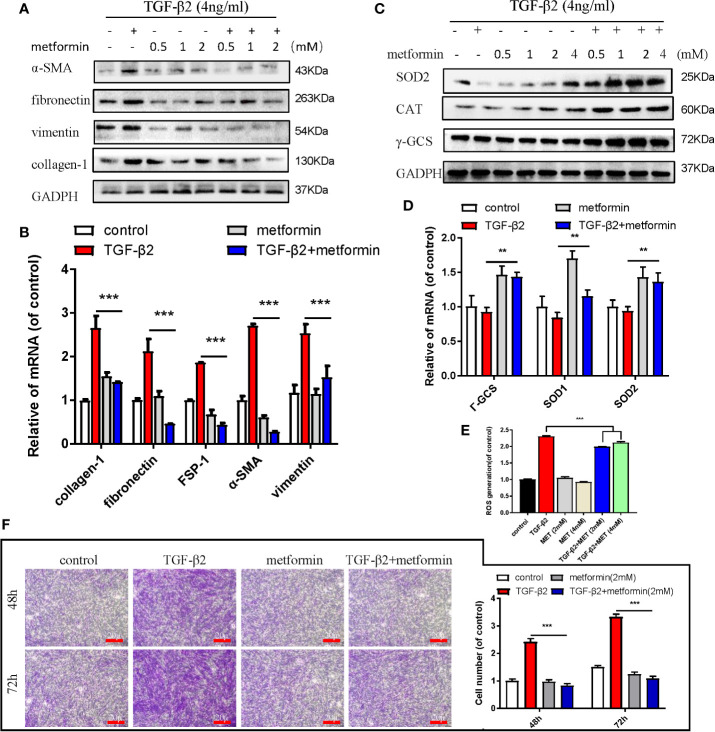
Metformin reduces TGF-β2-induced HConFs fibrosis. **(A**, **B)** The anti-fibrotic effects of metformin on TGF-β2 treated HConFs were analyzed by western blot/qRT-PCR. **(C**, **D)** The antioxidative effect of metformin on TGF-β2-induced HConFs was examined. **(E)** The ROS levels of HConFs were assessed by flow cytometry. **(F)** The migration capability of TGF-β2-induced HConFs followed by metformin treatment were analyzed by transwells and statistical analysis. n = 3. All data shown as mean values ± SD. ** and *** denote P < 0.01 and P < 0.001, respectively.

**Figure 4:**
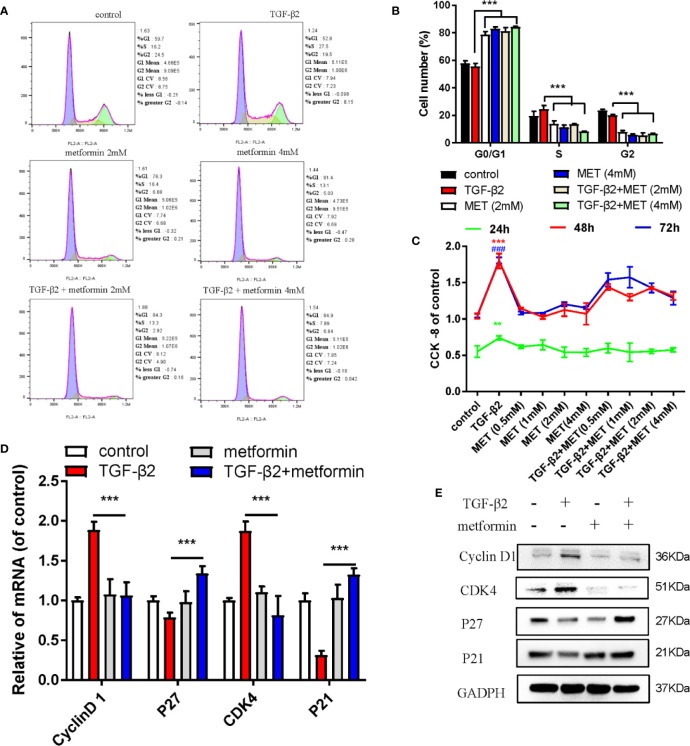
Metformin induces cell cycle arrest at G0/G1 phase in HConFs. **(A)** Cell cycle was analyzed by flow cytometry assay and **(B)** quantization of the rates in HConFs cells. **(C)** CCK8 was used to analyze the proliferation of HConFs. **(D)** The genes levels of CDK4, P21, CyclinD1 and P27 were assessed by qRT-PCR. **(E)** The relative cell cycle proteins level of CDK4, CyclinD1, P21 and P27 were tested by Western blotting. **p < 0.01, ^###/^** p < 0.001. MET = Metformin.

Metformin is also an AMPK activator; besides, it has been widely applied as a hypoglycemic agent in clinical, without any obvious complications (**Supplemental Figure S2A**). Here, we found that metformin could effectively reduce fibrotic and antioxidative relative proteins/genes level at a concentration of 2 mM in TGF-β2 treated HConFs followed by metformin treatment at different concentrations (**Figures 3A–D**). Similarly, metformin was as effective as AICAR (1 mM) in reducing increased ROS levels and decreased the migration of TGF-β2-induced HConFs (**Figures 3E, F**). To further analyze the role of metformin in HConFs proliferation, we found that metformin significantly inhibited cells proliferation in the G0/G1 phase by flow cytometry assay (**Figures 4A, B**). The CCK-8 assay results showed that metformin (2 mM) could effectively inhibit TGF-β2-induced cell proliferation (**Figure 4C**). Representative images showing immunofluorescence staining for fibronectin showed that AICAR or metformin effectively reduced related fibrotic levels (**Supplemental Figures S3A, B**). Furthermore, metformin reversed the expression of proliferative proteins and genes such as CyclinD1, CKD4, P21, P27 (**Figures 4D, E**), indicating that the activation of AMPK may promote antioxidant capacity and inhibits the cells migration in HConFs induced by TGF-β2. Therefore, the above results suggest that metformin, likes AICAR, may reduce TGF-β2-induced HConFs’ fibrosis, migration and proliferation by activating AMPK.

### Metformin Reverses Fibrosis in TGF-β2-Induced HConFs by Activating AMPK/Nrf2 Axis

**Figure 5:**
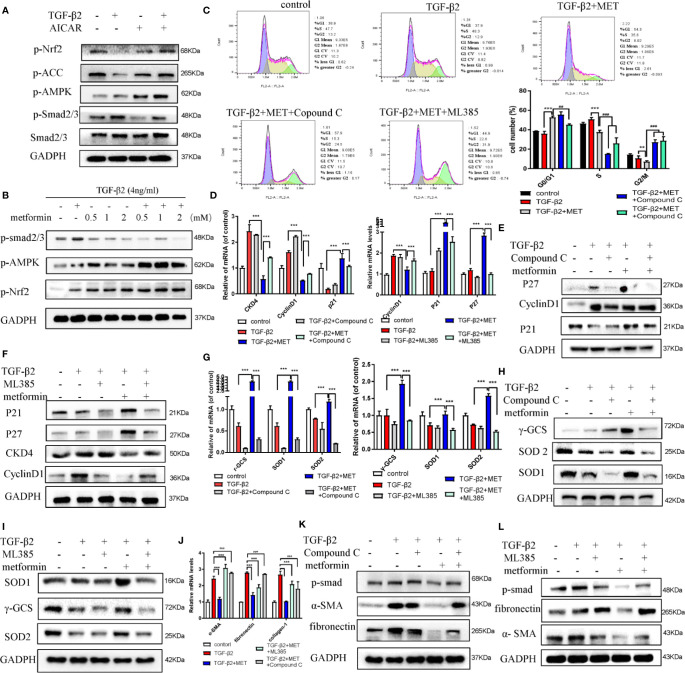
Metformin reduces fibrosis in HConFs pretreated by TGF-β2 *via* activating AMPK/Nrf2 axis. **(A**–**B)** AICAR or metformin could increase the levels of p-Nrf2(S40), Nrf2 and p-AMPK in HConFs after TGF-β2 precondition for 24 h. **(C)** Cell cycle was tested and quantization of the rates in HConFs cells. Compound C or ML385 could reverse the inhibition of metformin on HConFs’ proliferation. **(D**–**F)** Cell cycle relative proteins/genes (CDK4, CyclinD1, P21 and P27) were detected by qRT-PCR/western blotting. **(G**–**I)** Antioxidant defense relative protein/genes γ-GCS, SOD1/2 and **(J**–**L)** fibrotic proteins/genes collagen-1, fibronectin, α-SMA level in HConFs treated by metformin with/without Compound C (10 μmol/L) or ML385 (10 mM) treatment for 24 h. MET = metformin, n = 3. All data shown as mean values ± SD. **^/##^ and ***^/###^ denote P < 0.01 and P < 0.001, respectively.

Previous literatures reported that the activation of AMPK/Nrf2 pathway ameliorated hepatic damage *via* decreasing the inflammation, oxidative stress and fibrosis levels in T2DM and PA-induced oxidative stress and cell injury (Wang et al., 2017; Kim et al., 2019). Activating Nrf2 may increase the level of multiple transcription factors related to anti-inflammatory, antioxidants and other cell protection pathways by combining with antioxidant response elements (Shanmugam et al., 2017; Li et al., 2020). Here, we hypothesized that metformin increased the intracellular ROS scavenging capacity and inhibited proliferation of TGF-β2-induced HConFs by regulating the AMPK/Nrf2 signaling pathway. *In vitro*, we found that AICAR elevated the phosphorylation expressions of ACC and AMPK, but inhibited the level of p-smad2/3 (**Figure 5A**), and the effects of metformin at 2mM doses on HConFs induced by TGF-β2 was consistent (**Figure 5B**). On the contrary, silencing AMPK mediated the constitutive expression of fibronectin in cells after TGF-β2 treatment. Precondition with Compound C (an AMPK inhibitor) or ML 385 (a Nrf2 inhibitor) not only reversed metformin arrested cells in the G0/G1 phase (**Figures 5C–F**), but also decreased the capacity of metformin increased intracellular antioxidants degree on proteins and genes (**Figures 5G–I**). In **Figures 5J–L**, the relative fibrotic proteins and gens in TGF-β2-treated HConFs with pretreated by metformin were significantly increased followed by Compound C or ML 385 treatment. Together, the above experiments indicate that metformin reduces the levels of TGF-β2-induced HConFs oxidative stress, proliferation, fibrotic proteins/genes *via* activating AMPK/Nrf2 signaling pathway.

### Organic Cation Transporter (OCT) Mediates the Uptake of Metformin in HConFs Cell

**Figure 6:**
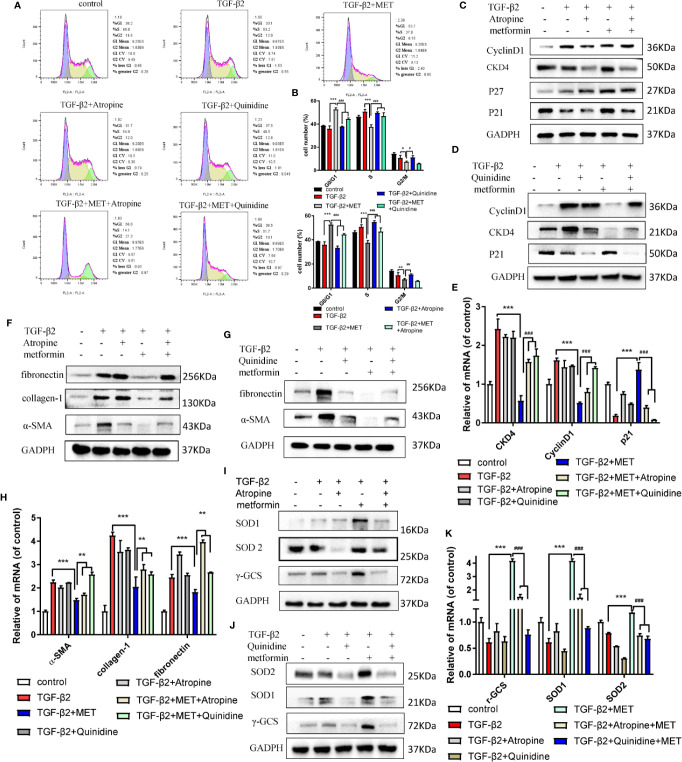
The inhibitor of OCTs could reduce the capability of anti-proliferation of metformin. **(A)** Cell cycle was assessed by flow cytometry assay and **(B)** quantization of the rates in HConFs. **(C**–**E)** Cell cycle relative proteins/genes (CyclinD1, CDK4, P21 and P27) level were assessed in HConFs after Atropine/Quinidine treatment by qRT-PCR and western blot. **(F**–**H)** Relative fibrotic proteins/genes were assessed. **(I**–**K)** Antioxidants related proteins and genes (α-SMA, collagen-1, fibronectin, N-cadherin and vimentin) were assessed in HConFs after Atropine/Quinidine treatment by qRT-PCR or western blotting. MET = metformin, n = 3. Data shown as mean values ± SD. *^/#^p < 0.05, **^/##^p < 0.01, ***^/###^p < 0.001.

Topical administration is the most convenient and patient compliant route of drug administration (Boddu et al., 2014). Metformin, a cation substrate of OCT, is one of few of the ophthalmic drugs that are transported by OCTs (atropine/quinidine), which reduce the uptake of cation substrates (Horie et al., 2011; Nirmal et al., 2012). For further study the effects of metformin on prolonging the survival of functional filtering blebs, the cation substrates of atropine/quinidine were used in this study. As shown in **Figures 6A, B**, atropine/quinidine significantly reversed metformin arrested HConFs at G0/G1 phase. The relative proteins and genes level of Cyclin D1, CKD4, P27 and P21 were reversed in TGF-β2-induced HConFs with metformin treatment followed by atropine/quinidine pretreatment (**Figures 6C–E**). At the same fibrotic and antioxidative proteins/genes level, atropine/quinidine significantly reduced the ability of metformin to resist fibrosis and improve antioxidant stress (**Figures 6F–K**). From the above results, we could conclude that the basis of metformin inhibit TGF-β2-induced HConFs fibrosis is transferring into cells by OCTs.

### Metformin May Reverse the Pro-Fibrosis Effect of Different Macrophages Polarization on HConFs

**Figure 7:**
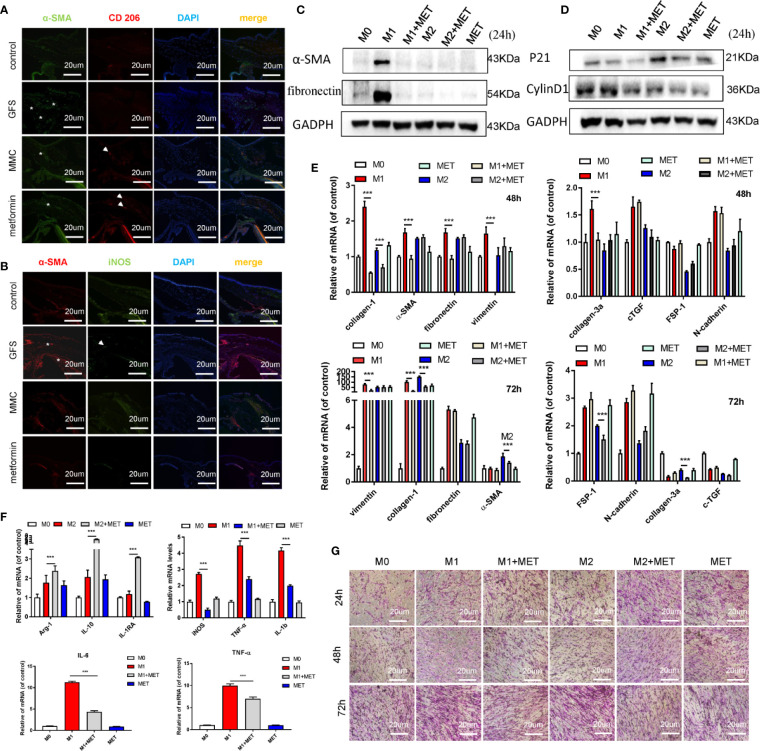
Metformin reduces the fibrotic levels of HConFs *via* regulating macrophage polarization. **(A**, **B)** Immunofluorescence co-staining of α-SMA and CD 206/iNOS in filtrating blebs tissues. **(C**–**E)** The fibrosis or proliferation relative genes and proteins level of HConFs co-cultured with macrophage. **(F)** Quantification of iNOS, IL-6, TNF-α and IL-1β in M1-polarized macrophages (or M2-polarized macrophages) with/without metformin precondition by qRT-PCR. **(G)** The migration capability of HConFs co-cultured with M0, M1- and M2-polarized macrophages followed by metformin treatment were analyzed by transwells. MET = metformin. Data shown as mean values ± SD. n = 3, ***p < 0.001.

In the process of fibrotic diseases, macrophages exerted bidirectional effects on regulating matrix deposition and resolution, which were also central players in tissues’ fibrosis (Ma et al., 2017; Conti et al., 2018; Han et al., 2018a). Macrophages could be polarized to the classical activation (M1) or alternative activation (M2) phenotype in the local immune microenvironment. Besides, ROS could damage the healing of skin wounds in the inflammatory stages through regulate the local microenvironments (Milich et al., 2019; Potter et al., 2019). Here, we found that topical application of metformin (7.85 mM/drops, four times/day) significantly inhibited the filtrating blebs scar formation in rats of GFS. There was a stronger immunofluorescence signaling of α-SMA and M1 macrophage phenotype in GFS group compared to the other group (**Figures 7A, B** **)** (as white stars/arrows showed). To study the fibrotic effects of metformin on macrophages to HConFs, the co-culture of macrophages of different polarities (M0, M1 and M2) and HConFs at different times were analyzed, the results showed that macrophages of different polarities showed different induction responses to HConFs at 24h (**Supplemental Figure S4A**), which were intricated and showed a lower level of fibrosis in general. Continue to observe co-culture for 48 and 72 h, we found that metformin reduced the fibrotic genes/proteins expression of HConFs in most M1 macrophages (induced by LPS of 100 ng/ml), and metformin could partially reduce the pro-fibrosis of HConFs cells by M2 macrophages (IL-4 + IL-13, 20 ng/ml) genes maker expression (**Figures 7C, E** **)**. Moreover, transwells assay showed metformin induced macrophage with IL-4 + IL-13 treatment increasing the cells migration in HConFs after co-culture 48 h. While co-culture 72 h, metformin reduced the cells migration and proliferation of HConFs (**Figures 7D, G** **)**. To know that metformin interfered with the induction of HConFs fibrosis by macrophages of different polarities, metformin was added simultaneously during the induction of different polarities of macrophages, and RNA samples were collected for 12 h. The data indicated that metformin reduced the phenotypic changes of M1 macrophages with pro-inflammatory effects, and decreased the genes level of some inflammatory factors. In addition, metformin also increased the expression of M2 macrophages with anti-inflammatory effects (**Figure 7F**). Moreover, metformin obviously promoted the migration of M0, M1 and M2, indicating that metformin could promote the capability of macrophages (**Supplementary Figure S4B**). These anti-inflammatory effects were supported by previous reports (Koh et al., 2018), suggesting that the anti-inflammatory capability of metformin reduced fibrosis in TGF-β2-induced HConFs. Besides, the results may provide a novel direction that metformin approve local microenvironments to increase success GFS rates.

## Discussions

Glaucoma is the leading cause of irreversible blindness and reduces the vision-related quality of life worldwide (Crish and Calkins, 2015; Williams et al., 2017). GFS is recognized as the most effective option for reducing IOP. However, intraoperative tissue damage may stimulate the conjunctival fibroblasts migration, proliferation and differentiate from fibroblasts to myofibroblasts (Lanzl et al., 2016) that is an important process of wound repair, but usually leads to scarring (Wick et al., 2013). Current clinical anti-conjunctival scar drugs such as MMC or 5-FU were widely applied to improve the outcomes of GFS, but are limited due to some serious complications (such as macular degeneration, filtered vesicle leakage and corneal epithelial dysfunction) (Cabourne et al., 2015). Thus, there is an urgent need for safer and more effective anti-scarring dugs in the clinic.

Accumulated studies have suggested that the effectively therapeutic role of metformin in non-diabetic diseases and has anti-fibrotic and anti-inflammatory effects (Faggi et al., 2018). Oral administration of metformin had protective effects on reducing in risk of developing OAG and had profound effects on anti-inflammatory and anti-angiogenesis on retinal vasculature (Lin et al., 2015; Han et al., 2018b). Besides, the transcorneal penetration of single topical administration metformin was significantly decreased with topical pre-treatment of OCT blockers (Atropine or Quinidine) (Wang et al., 2008). These studies provided evidences that a potential therapeutic effect of eye drops of metformin in patients with GFS. In this study, we found that eye drops of metformin could effectively extend postoperative filtrating blebs survival and reduced the inflammatory response of conjunctival flap in rats’ GFS model. Through immunohistochemical staining analysis of postoperative conjunctival tissues and surrounding vascular tissues, the expression of AMPK activation in damaged tissue was lower, while the protein of α-SMA was overexpressed than normal group. According to previous reports, the damaged tissues were affected by inflammatory factors such as platelet-derived growth factor PDGF, TGF-β and hormones induction inhibited the expression of AMPK, resulting in damaged cell metabolism, abnormal proliferation, and eventually scar formation (Mishra et al., 2008; Foo et al., 2011; Rangarajan et al., 2018). Whether metformin can inactivate differentiated fibroblasts and resolve fibrosis in HConFs are still unclear. In this study, as expected that AICAR activating AMPK could effectively inhibit the proliferation, migration and phenotypic transformation of HConFs in a stressed state after injury. Metformin, the other AMPK activator, also appeared to inhibit TGF-β2-induced HConFs oxidation and proliferation, migration, and fibrosis. In addition to the important role of fibroblasts in scar formation, macrophages also played key role in tissue remodeling. In this experiment, metformin not only directly regulated the process of HConFs fibrosis, but also reversed the pro-fibrosis effect of different macrophages polarization on HConFs by reducing phenotypic expression of M1 macrophages with pro-inflammatory effects. For further study the mechanisms of metformin prolonged the survival of filtration blebs, we hypothesized that metformin could reduce TGF-β2-induced HConFs by regulating the AMPK/Nrf2 signaling pathway to prolong the time of filtrating blebs. The proteins relevant to the AMPK/Nrf2 signaling pathway were assessed to test this hypothesis. Compound C (an AMPK inhibitor) and ML385 (a Nrf2 inhibitor) were used to verify the rationality of the pathway, As previous studies (Guo et al., 2018; Xie et al., 2020), the data indicated that Compound C/ML385 could effectively reverse the inhibition of metformin on the proliferation, migration and fibrosis of TGF-induced HConFs.

**Figure 8:**
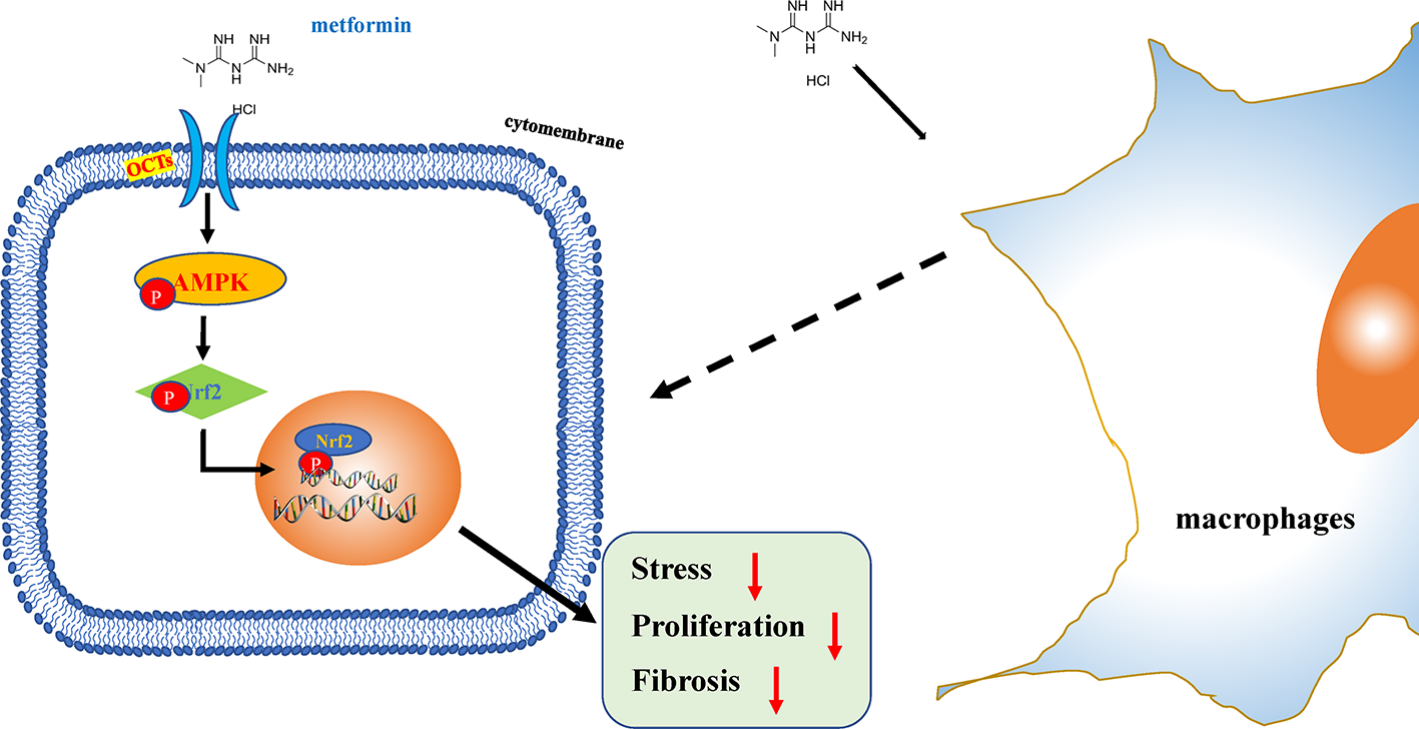
Schematic illustration of the mechanism of metformin reduces oxidative stress and decreases proliferation of HConFs against fibrosis. Briefly, metformin obviously promotes antioxidant defense, inhibits proliferation *via* activating AMPK/Nrf2 axis and the downregulation of inflammatory biomarkers to improves issues repair potential.

Eye, an organ with a special anatomical location, is convenient for local application while effectively avoiding the blood–eye barrier (Nirmal et al., 2013b). The absorption of drugs by cells can effectively improve the efficacy of drugs under certain conditions. Moreover, organic cation transporters (OCT) inhibitor drugs can inhibit drug effects by competing with OCT, indicating that the drug-drug interaction by inhibition of OCT transporters may be important. Metformin, known as a cation substrate of OCT, transported by the hepatic and renal depends on OCTs (Nirmal et al., 2013a). Through the application of atropine/quinidine in this study, we found that OCTs were important for metformin therapeutic action in response to anti-fibrosis. Metformin could not only potentially reduce fibrosis by increasing the antioxidant capacity, but also reduced the proliferation of injured HConFs, and reversed the pro-fibrosis effect of different macrophages polarization on HConFs (**Figure 8**). However, due to the scarcity of clinical secondary surgery specimens, the analysis of AMPK expression in clinical patients has been limited.

In conclusion, our data indicate that metformin has a significant anti-fibrotic effect on GFS rats, indicating that metformin eye drops should be regarded as a therapeutic option for patients with GFS progressive fibrosis.

## Data Availability Statement

The datasets generated for this study are available on request to the corresponding authors.

## Ethics Statement

The animal study was reviewed and approved by the National Institutes of Health and were approved by the Animal Care and Use Committee of Chongqing Medical University.

## Author Contributions 

XL and YL performed the experiments. XL, QJ, ZW, LC, CZ, HW, PL, XY, YW, LX, YZ and CS interpreted data and drafted the manuscript. XL and YZ performed the data analysis. XL, LX, ZW and CShi conceived and designed the study.

## Funding 

This work was supported by the (Chongqing) National Natural Science Foundation of China (Grant nos. 81670860, 81470629 and No. cstc 2018jcyjAX0034), University Innovation Team Building Program of Chongqing (CXTDG201602020, to CShi), National Key Research and Development Program (2016YFC1000805, to CShi).

## Conflict of Interest

The authors declare that the research was conducted in the absence of any commercial or financial relationships that could be construed as a potential conflict of interest.
