# Supplementary material for: Eye Drops of Metformin Prevents Fibrosis After Glaucoma Filtration Surgery in Rats via Activating AMPK/Nrf2 Signaling Pathway
Source: Front Pharmacol. 2020 Jul 31;11:1038. doi: 10.3389/fphar.2020.01038 (PMC7438907; doi:10.3389/fphar.2020.01038)
Supplement: Supplementary file 1 [file DataSheet_1.docx]

Supplementary Material


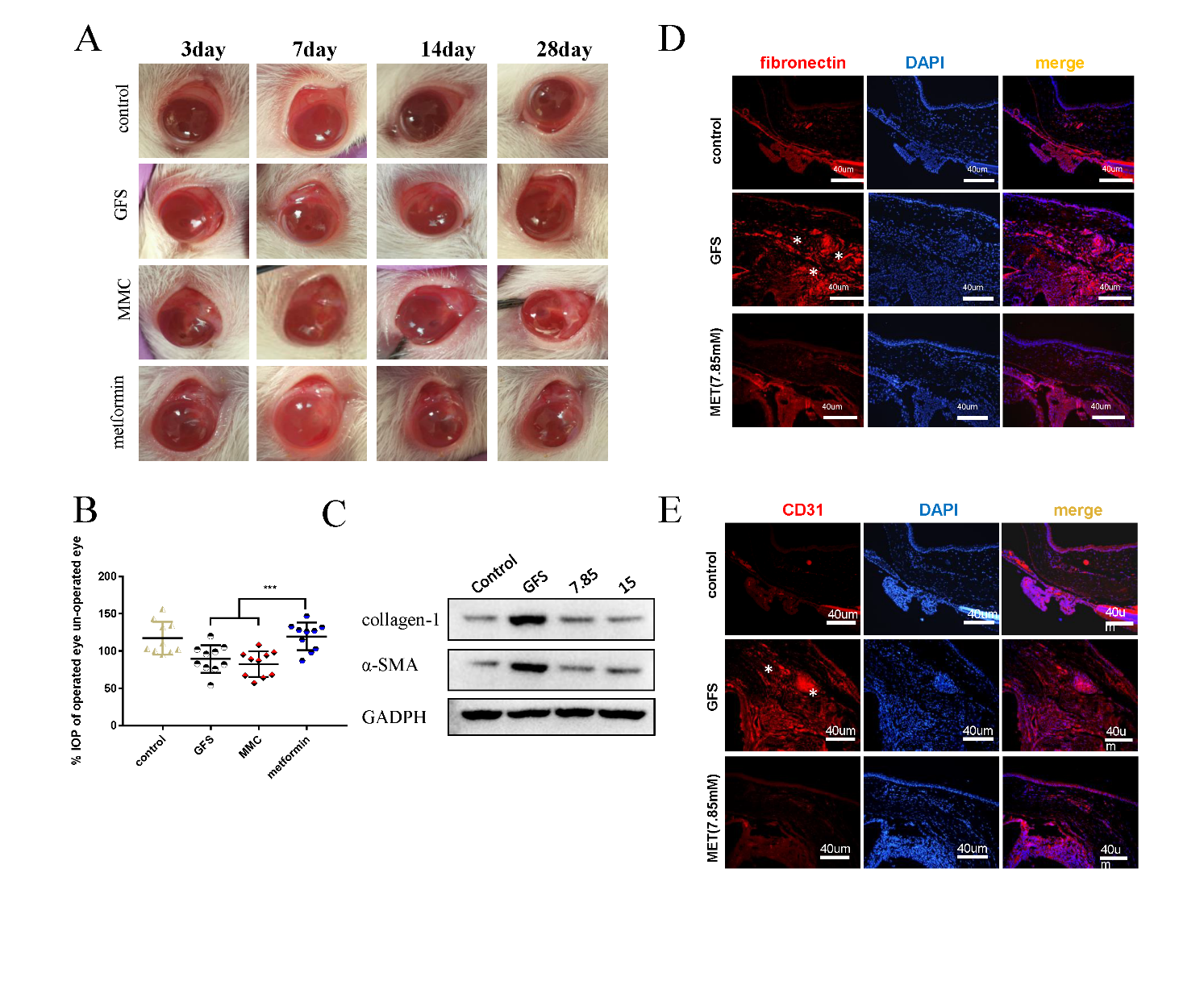


**Supplementary Figure 1.** (A) Representative stereo-microscopic images captured showing bleb characteristics in different groups of rabbits’ eyes at day 3, 7, 14 and 28 after GFS. (B) IOPs of the operated and un-operated eyes were measured. The numbers indicated the mean IOP percentage of control group (n = 5). (C) Fibrotic protein levels of α-SMA was examined by western blotting subconjunctival fibrosis in rabbits of postoperative 28 day. (D and E) The fibrotic levels of filtrating blebs were analyzed. Representative images showing immunohistochemical staining for fibronectin and CD 31 in ocular tissue sections, collected at day 14 after GFS. (Nuclei = blue, red = fibronectin/CD 31).


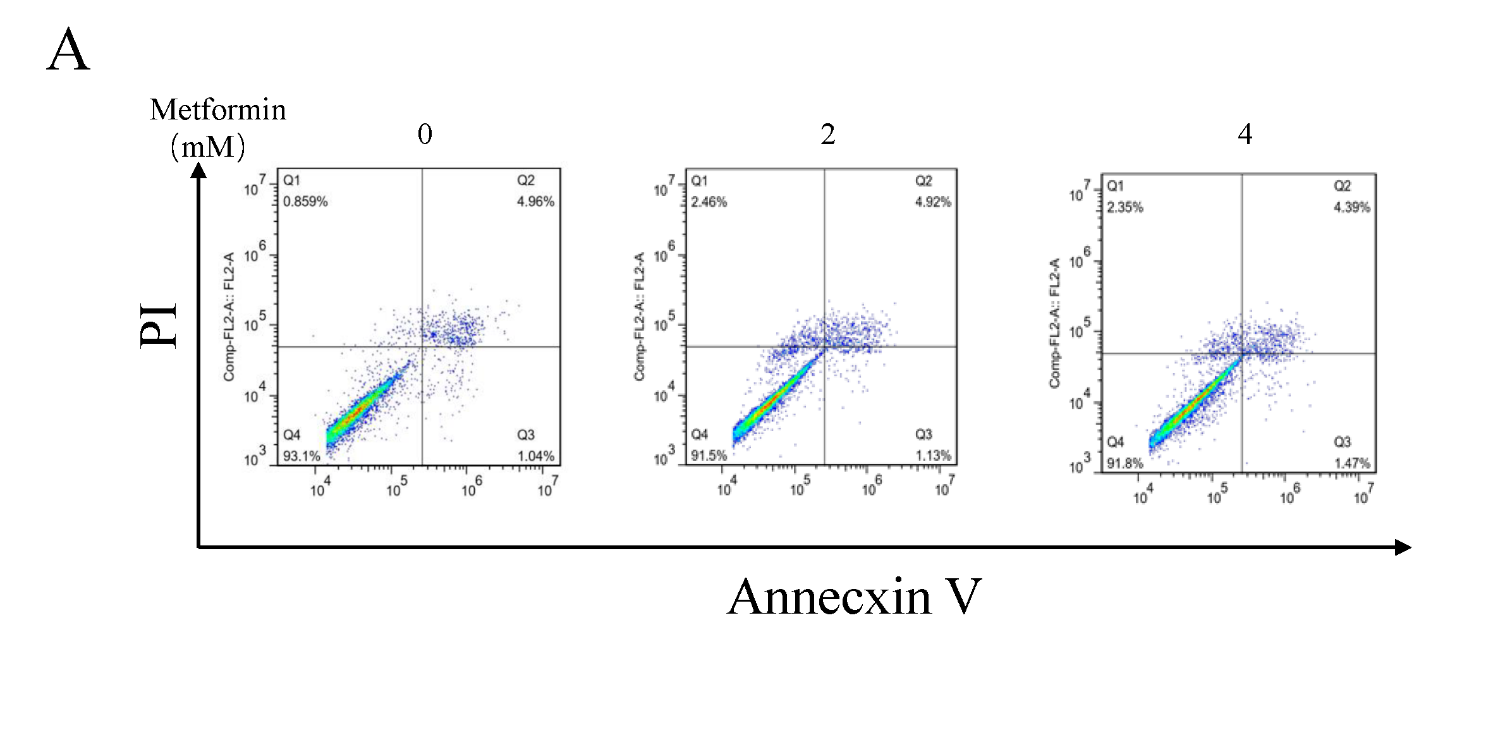


**Supplementary Figure 2** (A) The apoptosis rate in HConFs treated with different concentrations of metformin (0, 1 or 2mM) for 24 h, was analyzed by flow cytometry using Annexin V- PI.


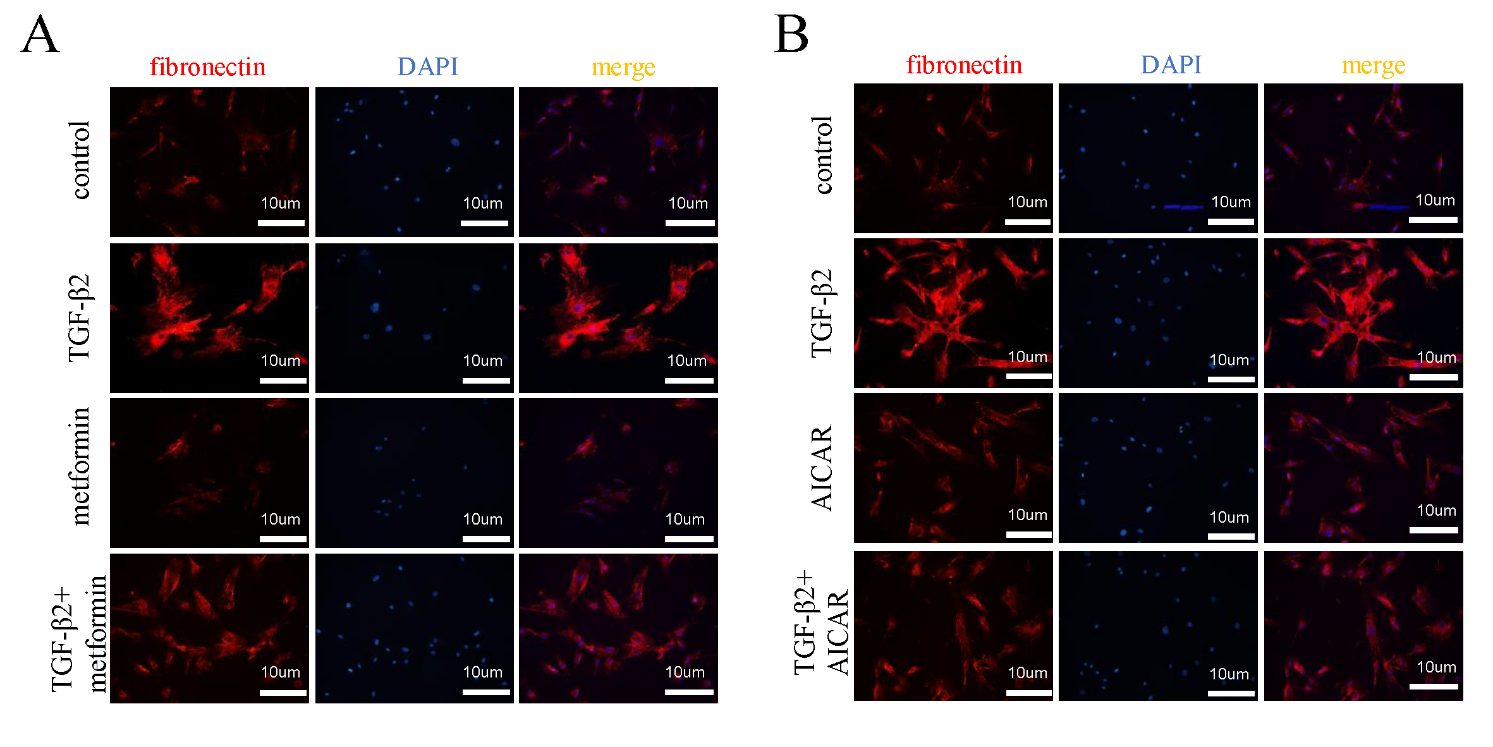


**Supplementary Figure 3 (**A-B) Representative images showing immunofluorescence staining for fibronectin generated after preconditioning with TGF-β2 followed by AICAR or metformin (Nuclei=blue, fibronectin=red)


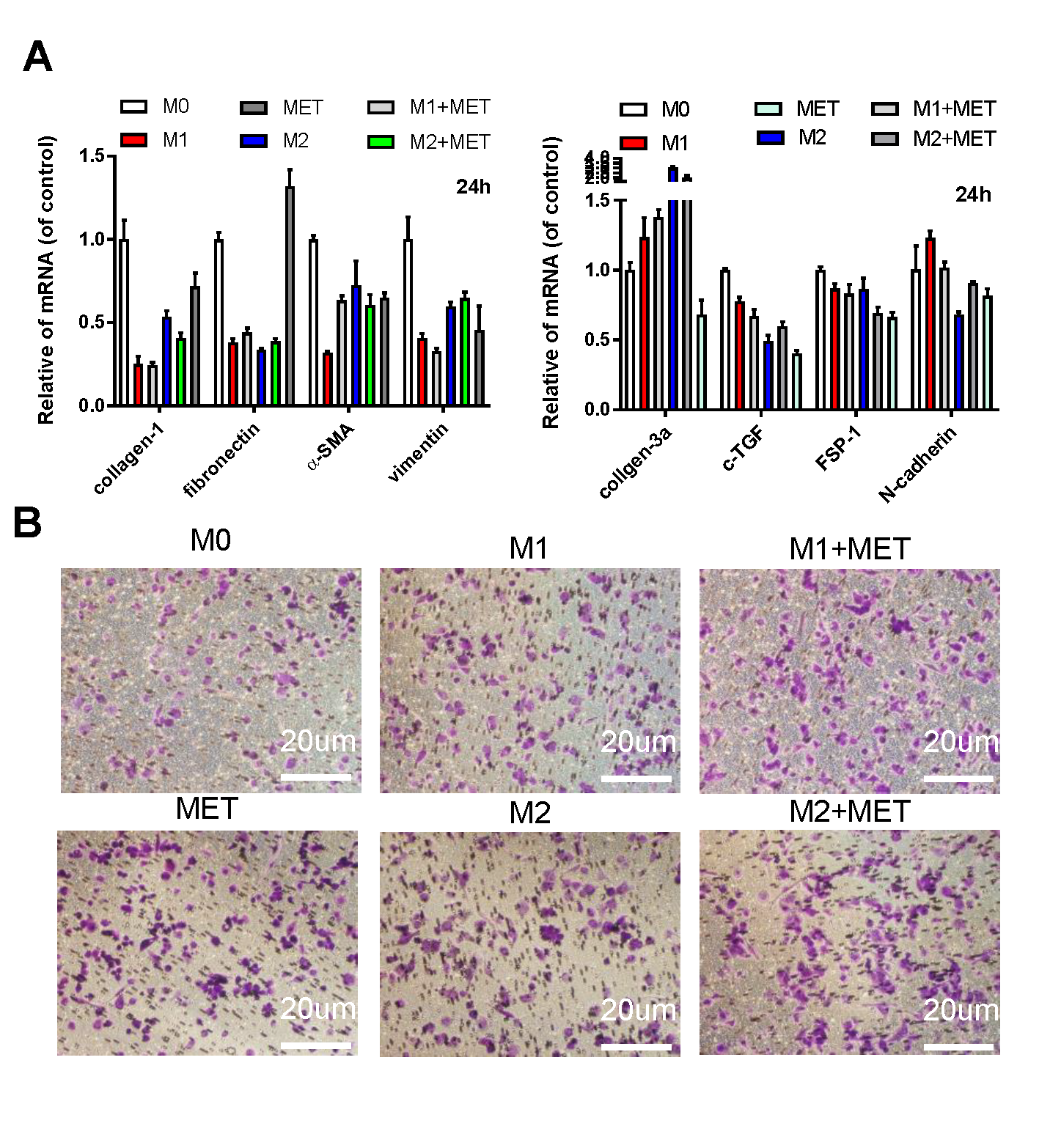


**Supplementary Figure 4 (**A) The fibrosis related genes level of HConFs co-cultured with macrophage at 24h. (B) The migration capabilities of M0, M1- and M2-polarized macrophages followed by metformin treatment were analyzed by transwells. MET=metformin, n = 3. Data indicate the mean ± SD. *p < 0.05, **p < 0.001.
